# Posterior sclerectomy for persistent serous retinal detachment with secondary glaucoma in Sturge–Weber syndrome: A case report

**DOI:** 10.1097/MD.0000000000034144

**Published:** 2023-06-30

**Authors:** Ariyanie Nurtania, Yoshiaki Kiuchi, Aisyah Muhlisah, Kazuyuki Hirooka, Naoki Okada, Hiromitsu Onoe, Kana Tokumo

**Affiliations:** a Department of Ophthalmology and Visual Science, Graduate School of Biomedical Sciences, Hiroshima University, Minami-ku, Japan; b RS Mata Makassar, Ministry of Health, Makassar, Indonesia; c RS Andi Sultan Dg Raja Bulukumba, South of Sulawesi, Indonesia.

**Keywords:** case report, proton beam therapy, scelerectomy, serous retinal detachment, Sturge-Weber syndrome

## Abstract

A serous retinal detachment is one of the most likely ocular manifestation of Sturge-Weber syndrome (SWS). This finding can frequently occur as a complication after filtering surgery to maintain the intraocular pressure (IOP). Proper treatment has been approached with choroidal hemangioma as an organ target. To the best of our knowledge, various treatments for SRD are associated with diffuse choroidal hemangioma has been approached. However, a second retinal detachment following radiation therapy has worsened the situation. Here, we report an unexpected serous retina and choroidal detachment after non penetrating trabeculectomy. Although radiation therapy has been proposed for previous detachment to the ipsilateral eye in this case, repetition radiation therapy was not suggested concerning the health and quality of life especially in for young subjects. However, the kissing choroidal detachment in this case necessitated immediate intervention. Thus, posterior sclerectomy was performed for the recurrent retinal detachment. We believe that an intervention for a SWS case related complication will remain a significant and important to share as a public health contribution.

**Case presentation:**

A 20 year-old male confirmed with SWS with no known family history was diagnosed with SWS. He was gain from another hospital for glaucoma therapy. On the left brain MRI showed severe hemiatrophy in the frontal and parietal lobes and leptomeningeal angioma. Although his right (RE) eye had 3 gonio surgeries, 2 Baerveldt tube shunts and *Micropulse trans-scleral cyclophotocoagulation,* his IOP remained uncontrollable when he was 20 years old. RE IOP was in controlled after non-penetrating filtering surgery hence, his RE developed a recurrent serous retinal detachment. A posterior sclerectomy was performed in 1 quadrant of the globe to drain subretinal fluid.

**Conclusion:**

Sclerectomies to the inferotemporal quadrant of the globe for serous retinal detachment associated with SWS are considered efficient for optimal drain subretinal fluid, resulting in complete regression of detachment.

## 1. Introduction

Sturge-Weber syndrome (SWS) is a congenital, non-hereditary disorder that is characterized by a facial port-wine stain, affects approximately 1 in 20,000 to 50,000 live births, and is related to a somatic mutation in the GNAQ gene.^[1]^ The classical facial port-wine stain, involving the branch of the trigeminal nerve and the embryonic vasculature distribution in this area, induces certain ophthalmic complications of the anterior segment entailing the eyelids and conjunctiva. In patients with SWS, the choroid is one of the most important sites of vascular adjustments and can be easily seen as a typical “tomato ketchup” appearance.^[2]^

Previous studies have shown that increased choroidal thickness in SWS patients may lead to severe retinal complications such as subretinal fluid (SRF), retinal degeneration, serous retinal detachment, tortuous retinal vessels, and optic disc coloboma, which contribute to visual loss and visual field defects.^[3]^ Hence, SWS is typically associated with diffuse choroidal haemangioma that may present first as glaucoma or amblyopia in children.

Serous retinal detachment can be controlled with various therapies. There are several methods, such as internal beta blocker use, low power infrared laser therapy, photodynamic therapy, and radiation therapy, including external beam radiation therapy, proton beam irradiation, stereotactic radiotherapy, and plaque radiotherapy.^[4]^

We experienced recurrent serous retinal detachment in a young patient with SWS after intraocular pressure (IOP) lowering therapy. Repetition radiation therapy was a contraindication for health-related quality of life in patients with previous serous retinal detachment who received proton beam therapy. We describe a posterior sclerectomies surgery that appears to be efficient in eliminating SRF immediately in a patient with SWS who underwent proton beam therapy.

## 2. Case presentation

We followed up a 20 year-old male with SWS at Hiroshima University Hospital since 10 years ago. A review of the medical history of his family members revealed no known medical history of SWS. He was gain from another hospital for an uncontrolled IOP. At baseline examination, port-wine stain showed bilateral frontal and maxillary distribution, and the best corrected visual acuity was 20/50 with +1.25DS −1.0DC A90 in the right eye (RE) and 20/40 with +4.50DS −1.25 A25 in the left eye (LE). The IOP was 27 mm Hg in the RE, and 24 mm Hg in the LE with 4 anti-glaucoma eyedrops. The anterior chamber depth was shallow in the RE and normal in the LE, and mild lens opacity in both eyes. He had a history of undergoing an MRI procedure that showed severe left-brain hemiatrophy in the frontal and parietal lobes and leptomeningeal angioma.

**Figure 1.**
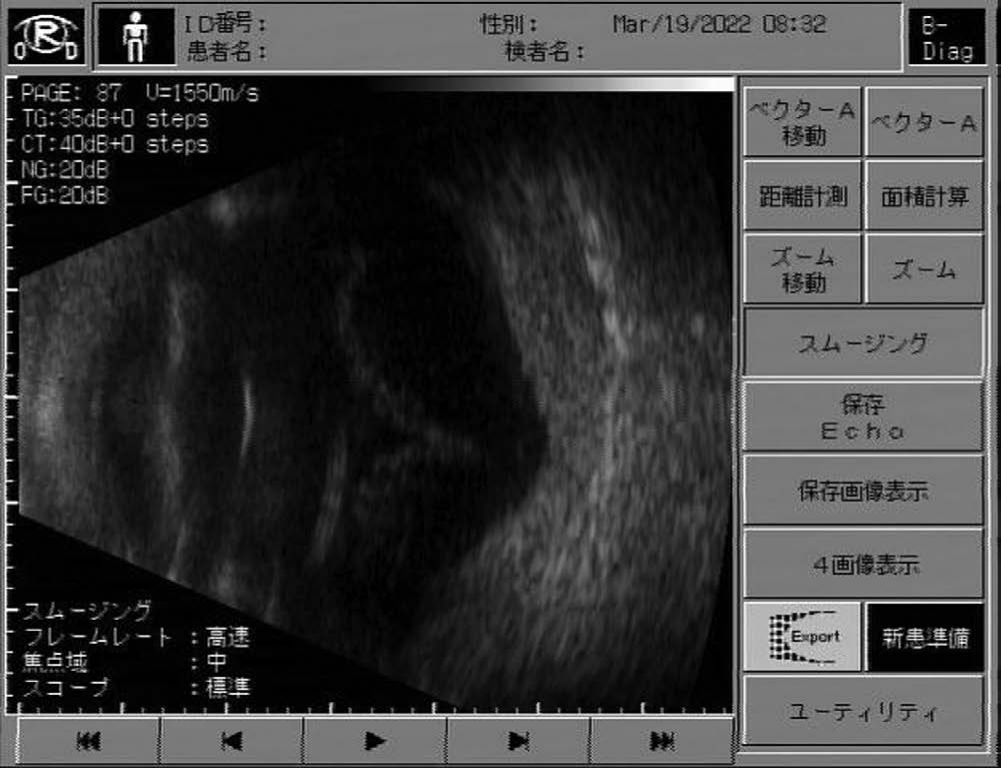
Fundus photography of the right eye. A. Preoperative fundus photography of the right eye revealed a smooth dome-shaped appearance from the nasal to the temporal region with serous retinal detachment. B. Postoperative day 2, the retina had reattached and the serous retinal fluid has resolved.

**Figure 2.**
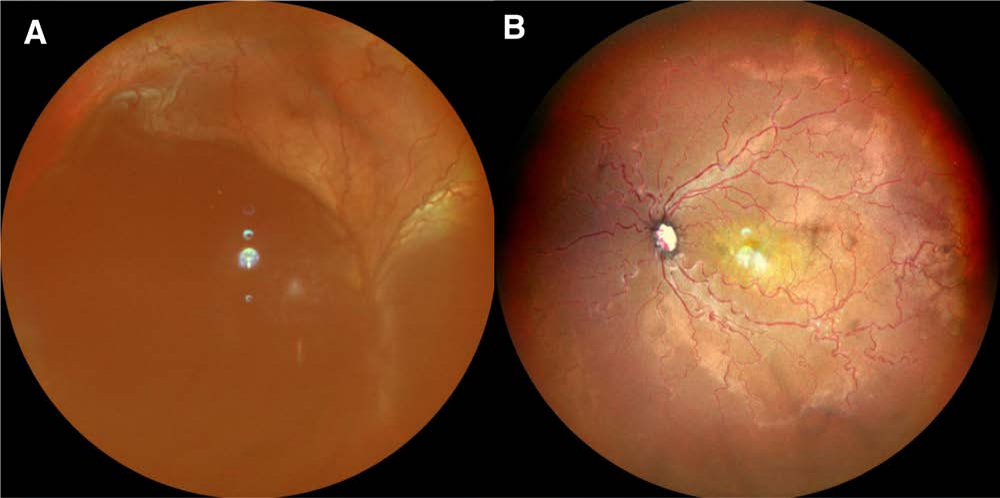
B-mode ophthalmic ultrasound. Showed retinal detachment and kissing choroidal from appositional serous choroidal detach.

Has undergone multiple surgeries since birth, and in 2015, his RE developed serous retinal detachment. Intravitreal injection of anti-vascular endothelial growth factor (VEGF) combined with photodynamic therapy (PDT) was ineffective, whereas proton beam therapy has successfully reattached the retina. Previous surgeries to decreased IOP included 3 trabeculotomies in both eyes and a Baerveldt tube shunt in the RE in 2018. *Micropulse trans-scleral cyclophotocoagulation* procedure in both eyes was performed in 2020 and 2021. In early 2022, IOP in the RE increased to nearly 30 mm Hg. We performed non penetrating trabeculectomy on March to the RE with an IOP monitoring outcome between 15 and 20 mm Hg. Severe retinal and choroidal detachments developed at the end of March 2022 (Figs. 1 and 2).

To manage this situation, we prescribed acetazolamide 500 mg/d to activate the retinal pigment epithelium and to decrease the transudation of choroidal vessels, and performed scleral window surgery in the inferior temporal quadrant of the globe to support transscleral drainage. As a result, drainage of the choroidal fluid allows retinal reattachment and enhanced uveoscleral outflow.^[3]^ Two days after the drainage procedure, the SRF vanished, allowing the retina to reattach and improve the uveoscleral outflow.^[3]^

Informed consent is provided by our patient who is aware of this case report. The data was also acquired with the ethical consent of Hiroshima University.

## 3. Discussion

After the filtering surgery, a serous retinal detachment occurred in a 20-year-old male with SWS. A history of prior retinal detachment was treated with proton beam therapy 7 years ago, while recurrent retinal detachment was treated with acetazolamide administration and a posterior sclerectomy surgery.^[5]^

Treatment of serous retinal detachment must address the underlying local or systemic disease which in this case choroidal haemangioma has been a major causes.^[6]^ Conventional therapy includes photodynamic therapy, external beam radiation therapy, and oral propranolol.^[7]^ PDT produces selective occlusion of vascular lesions with minimal harm to the adjacent retina, allowing the treatment of sub foveal choroidal haemangioma which has better results for Circumscribed choroidal hemangiomas than other treatment. Unfortunately, there is a potential that PDT will result in choroidal atrophy, which results in significant visual loss. Furthermore, choroidal hemangiomas can secrete VEGF, and PDT itself has been documented to cause a high level of VEGF expression.^[7]^ In our case the previous anti-VEGF injection combined with PDT procedures was ineffective. In patients with extremely thick choroidal hemangiomas and extensive non rhegmatogenous retinal detachment that fails to respond to PDT, low dose ocular irradiation appears to be an effective option.

The advantage of proton beam irradiation therapy is that a homogeneous dose can be delivered to the target while sparing the healthy tissue surrounding the tumor.^[8–10]^ It precisely delivers the radiation to the target tumor tissue by protecting the others near the ocular structures. proton beam irradiation therapy causes fewer radiation complications such as cataracts, radiation papillopathy, or retinopathy than external beam radiation therapy.^[11]^ However, the risk of developing a radiation-induced secondary cancer, particularly among young patients, is of concern in radiation therapy as the number of cancer survivors with a long post treatment life is increasing. Tsimhoni et al,^[12]^ reported that young adults and pediatric patients tended to receive higher secondary organ doses than adults due to a geometrical factor.

Surgical management of glaucoma in SWS is greatly challenging. IOP manipulation can change the permeability of vessels in large choroidal hemangiomas. Here, when we decreasing IOP results in expansion of the choroidal hemangioma with effusion of fluid into the suprachoroidal and subretinal spaces.^[4]^ A high choroidal vascular pressure is the result of open connection between the arteriolar system and choroidal vasculature without any intervening vascular bed. The high pressure and IOP conditions were reversed. When the IOP is reduced after the filtration surgery, the high choroidal vascular pressure causes choroidal effusion, increased fluid in the eye goes toward the sub-retinal space and causes retinal detachment.^[13]^ Prohylactic sclerotomies have been advocated, to be performed prior to ocular decompression, during filtering procedures in order to avoid these complications.^[14]^ Tsimhoni et al^[12]^ questioned the need for sclerotomy after investigating 17 eyes undergoing trabeculectomy.

**Figure 3.**
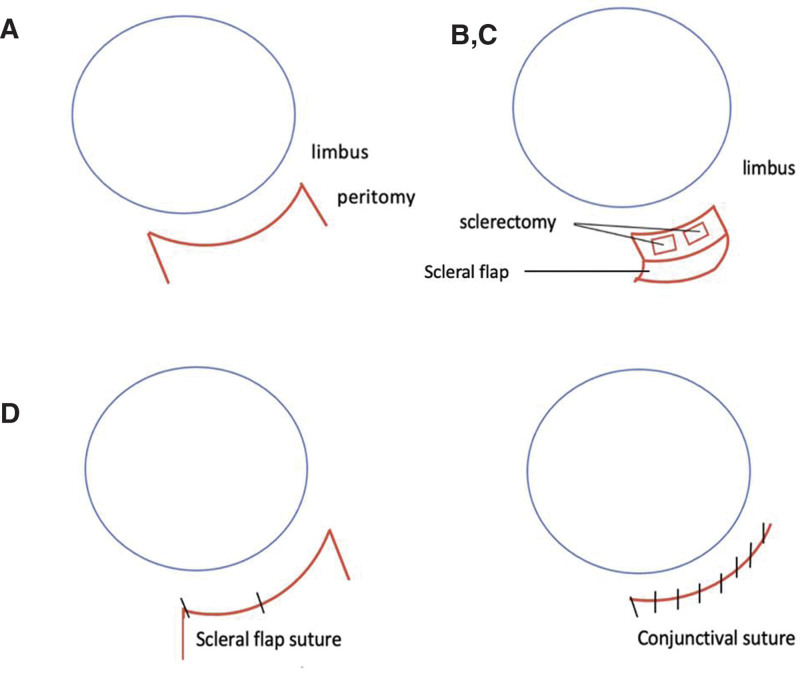
Schematic of sclerectomy procedure. A. Fornix-based conjunctival opening. B. Creating a 4 × 4 mm superficial half thickness scleral flap at 8 mm from the limbus between the inferior rectus muscle and lateral rectus muscle. C. Under the scleral flap, 2 small sclerostomies directly to the choroid were excised with a blade subretinal fluid was drained through the sclerostomy and was left open to support additional drainage post operatively. D. The scleral flap closed with 2 sutures and conjunctiva are closed with tight interrupted 10-0 nylon sutures.

In our case, the fluid was non-resolving and retinal detachment was chronic and bullous, permanent damage to the retinal pigment epithelium as well as outer retinal structures occurs and persistence of subretinal fibrin may have lead to subretinal fibrotic scar formation. Aggravated with kissing choroidal resulted in an immediate need for effusion drain intervention required immediately. To avoid these complications, surgical intervention is planned under oral Acetazolamide cover 500 mg/d to activate the retinal pigment epithelium and to decrease the transudation of choroidal vessels.^[15]^ On pre-operative examination with supine position funduscopy, the main fluid was found in the inferior site of the globe (Fig. 3). A Scleral window surgery was made with a lamellar scleral flap and 2 tiny scelerectomies in the inferior lamellar quadrant, were left open during a scleral window operation to facilitate transscleral drainage and allow for further drainage even after the surgery. Here, we just used one of the 4 quadrants of the globe to ensure space for future decision. Larger case series with similar parameters and longer follow up are required to further elaborate the role of such procedures.

## 4. Conclusions

As repeated radiation therapy is not recommended for young patients, posterior sclerectomy surgery may be considered as a surgical option for the management of recurrent serous retinal detachment in patients with SWS subjects especially those who have undergone radiation therapy for previous serous retinal detachment. Larger case series with similar parameters and longer follow-up periods are required to further elaborate on the role of such procedures.

## Author contributions

**Conceptualization:** Ariyanie Nurtania, Yoshiaki Kiuchi.

**Data curation:** Naoki Okada, Kana Tokumo.

**Investigation:** Ariyanie Nurtania, Aisyah Muhlisah, Kana Tokumo.

**Methodology:** Ariyanie Nurtania, Kana Tokumo.

**Supervision:** Yoshiaki Kiuchi, Kazuyuki Hirooka.

**Validation:** Ariyanie Nurtania, Aisyah Muhlisah, Naoki Okada.

**Writing – original draft:** Ariyanie Nurtania.

**Writing – review & editing:** Ariyanie Nurtania, Naoki Okada, Hiromitsu Onoe.
